# 3D Bioprinting for Pancreas Engineering/Manufacturing

**DOI:** 10.3390/polym14235143

**Published:** 2022-11-25

**Authors:** Yukun Xu, Dabin Song, Xiaohong Wang

**Affiliations:** Center of 3D Printing & Organ Manufacturing, School of Intelligent Medicine, China Medical University, Shenyang 110122, China

**Keywords:** 3D bioprinting, organ engineering/manufacturing, vascularization, bioartificial pancreas, stem cells

## Abstract

Diabetes is the most common chronic disease in the world, and it brings a heavy burden to people’s health. Against this background, diabetic research, including islet functionalization has become a hot topic in medical institutions all over the world. Especially with the rapid development of microencapsulation and three-dimensional (3D) bioprinting technologies, organ engineering and manufacturing have become the main trends for disease modeling and drug screening. Especially the advanced 3D models of pancreatic islets have shown better physiological functions than monolayer cultures, suggesting their potential in elucidating the behaviors of cells under different growth environments. This review mainly summarizes the latest progress of islet capsules and 3D printed pancreatic organs and introduces the activities of islet cells in the constructs with different encapsulation technologies and polymeric materials, as well as the vascularization and blood glucose control capabilities of these constructs after implantation. The challenges and perspectives of the pancreatic organ engineering/manufacturing technologies have also been demonstrated.

## 1. Introduction

**Figure 1:**
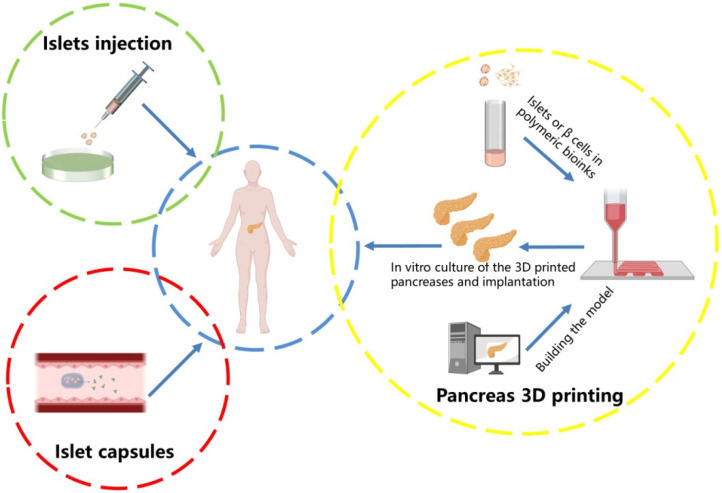
Diabetes treatment strategies. The traditional treatment for Type 1 diabetes is to inject insulin or take islet capsules. With the development of science and technologies, 3D printing implantable pancreatic organs is becoming more and more popular.

Diabetes is caused by a fault in the insulin production of the body and has different types. Type 1 diabetes mellitus is a common chronic disease in which the human immune system constantly attacks and destroys β cells, leading to insufficient insulin supply or insulin resistance, further causing the rise of blood glucose levels [1]. The main feature of type 2 diabetes is insulin resistance. The pancreas can produce insulin, but human organs no longer fully respond to insulin. As a result, with the continuous increase in insulin production, the overall β cell function and quality decline distinctively [2]. Currently, patients with type 1 diabetes can be controlled by injecting insulin and taking drugs to alleviate the disease (Figure 1). Type 2 diabetes is mainly controlled by diet and movement. However, diabetes can lead to irreversible tissue and organ damage with a variety of life-threatening secondary metabolic syndromes (MS), including neuropathy, retinopathy, nephropathy, stroke, and heart failure [3].

Clinical trials over the past few decades have demonstrated that pancreatic islet transplantation is an effective treatment [4,5]. Nevertheless, this therapeutic approach has been greatly limited by the shortage of islet donors and the low survival rate of the transplanted islets. Meanwhile, the allogeneic immune response of the transplanted islets can cause tissue rejection and further death of the transplanted cells. The need for lifelong immunosuppressants has also significantly restricted the widespread of this therapeutic approach [6,7].

It is recognized that the use of immunosuppressive drugs can cause a variety of serious adverse effects, such as nephrotoxicity, liver toxicity, and other abnormalities [8]. Over the last two decades, many researchers turned to wrapping the islets with biocompatible polymers as an immunoprotective barrier [9]. However, the traditional encapsulation methods have numerous shortcomings, such as hypoxia, lack of blood supply networks, and difficulty in the degradation of the polymers [10]. Without proper vascular networks, most of the transplanted cells disappear in the body or die quickly inside the capsules [11].

Parallelly, the traditional tissue engineering approaches for bioartificial tissue and organ engineering/manufacturing have been totally substituted by three-dimensional (3D) bioprinting technologies. Typical 3D bioprinting technology is characterized by printing cells, growth factors, hydrogels, and other biomaterials as ‘bioinks’ to produce bioartificial tissues and organs through automatic layer-by-layer deposition processes under the guidance of computer-aided design (CAD) models. Different types of cells can be encapsulated in different polymeric ‘bioinks’ and deposited simultaneously through multi-nozzle 3D printers. The hydrogels can absorb and retain large amounts of water, which is beneficial for cell growth, proliferation, differentiation, and tissue/organ formation [12]. The advanced 3D bioprinting technologies represent a high potential for pancreas constructions and type 1 diabetes therapies.

## 2. Pancreatic Islets and β-Cells

**Figure 2:**
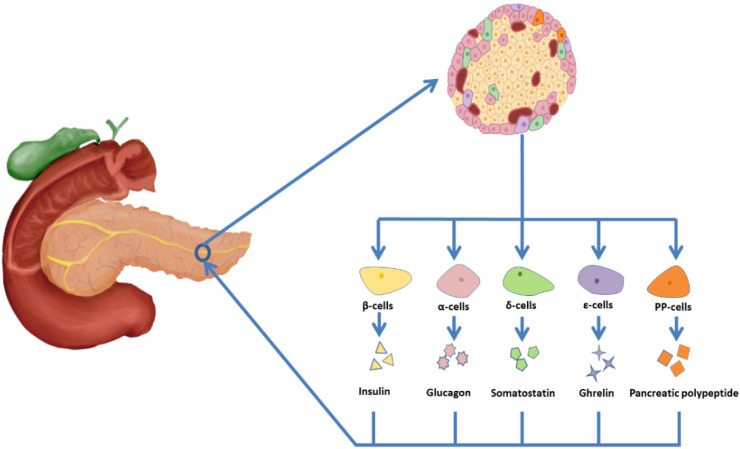
Islet cell composition and secretions. Islet cells are generally divided into β cells (50–60%), which secrete insulin, α cells (30–45%), which secrete glucagon, δ (less than 10%) cells, which secrete somatostatin, PP cells (about 1%), which secrete pancreatic polypeptide, and ε cells (less than 1%), which secrete growth hormone.

In the human body, the adult pancreas is a heterogeneous gland consisting of an exocrine chamber and an endocrine chamber. The exocrine part of the pancreas consists of secretory cells that produce digestive enzymes and release them into pancreatic ducts. The endocrine part consists of islets, which produce hormones and regulate glucose homeostasis (Figure 2). There are about 1.5 million islets in the pancreas. In each islet, 50–60% of the cells are β-cells, which secrete insulin, 30–45% are α-cells secreting glucagon, less than 10% are δ-cells secreting somatostatin, about 1% are pancreatic polypeptide cells (PP-cells) secreting pancreatic polypeptide, and less than 1% are ε-cells secreting ghrelin [13,14].

β cells are so special that half of the protein they create can be converted into insulin. The newly translated insulins can package into small particles. The insulin particle is an organelle in which many regulatory pathways intersect, which acts as the origin of several signals to regulate the activities of β cells. The changes of β cell activities in the plasma membrane directly result in the changes of glucose concentrations in blood with the stimulation or inhibition of secretions of insulins [15,16].

## 3. Islet Encapsulation

For nearly 20 years, cell encapsulation techniques have been explored to protect the transplanted heterospecific cells from the host immune system. The principle of islet encapsulation is to engraft cells into compartments separated by a semipermeable polymer membrane of capsules. The capsules can protect the islets from damage caused by the immune response. In addition to the protective mechanism provided by the capsules, the islets in the capsules can regulate blood sugar levels by releasing insulin, while small molecules (e.g., glucose and nutrients) and metabolic wastes can pass through the semipermeable polymeric membrane. Therefore, the encapsulation system can be considered as a ‘mini-bioartificial pancreas’, ‘micro-organ’, or ‘organoid’ [17,18].

However, the progress of cell encapsulation is very slow. At present, only a few cell encapsulation techniques have been applied in clinical trials with little therapeutic effects. Due to the complexity of cell encapsulation techniques, the immune response generated by the components (e.g., polymer materials, embedded cells, foreign genes, and genetically engineered DNA vectors) has not been effectively exempted. Although the use of high-purity polymers can reduce the immune response to some extent, more and more researchers believe that reducing the immunogenicity of the cells inside the microcapsules is the key to preventing the immune rejection of the transplants.

It is generally believed that the ideal polymers for islet cell encapsulation should be completely inert (no immune rejection), non-degradable (exist in the body for a long time), highly compatible with the encapsulated cells (maintaining cell survival and function), and have a smooth surface and strong hydrophilicity (reduce protein and cell attachment) [19]. To improve the properties of the encapsulation polymers, many methods, such as changing the chemical composition of the encapsulating materials, and co-encapsulating immunomodulators, have been exploited [20]. It is expected that after the microcapsules are transplanted into the recipients, new blood vessels should be established immediately to maintain the survival of the islets [21]. In past studies, nearly all the islet encapsulation techniques employ natural polymer, such as alginate, to encapsulate islets, since alginate has a certain degree of biocompatibility and can be cross-linked by divalent cations [22,23]. Nowadays, some synthetic hydrogels and their derivatives have been employed in islet encapsulation, since these synthetic hydrogels outperform natural hydrogels with respect to tunable properties, such as porosity, stability, mechanical strength, and biocompatibility [24].

As the goal of islet xenotransplantation is to restore insulin secretion in the recipients, a large number of islets is a prerequisite to ensure that the transplantation has enough living cells. When sufficient insulin is secreted by the transplanted islets, the sugar level of the blood can be controlled efficaciously. At the same time, oxygen and nutrient supplies for the grafts should be kept up with [25,26]. In order to ensure the stability of the transplants, rapid vascularization is desired, to maintain the insulin secretion function under the stimulation of glucose.

A typical islet encapsulation technique was described in 2009 by Zhang et al. [27]. In this study, a non-adhesive islet encapsulation layer based on synthetic polyethylene glycol diacrylate (PEGDA) was used as the first layer. To increase the vascularization effects, thiogelatin, thioheparin, and thiohyaluronic acid were used as the second layer to provide endothelial cell adhesion points and act as a growth factor release matrix [28]. The PEGDA coatings can be covalently applied on the surface of islets, and the islets can be subsequently embedded in hydrogels containing thioglycosaminoglycans. Experiments have shown that this method can effectively control the release of growth factors, promote the growth of blood vessels to the embedded islets, and maintain the shape and function of the islets after implantation [29].

Another typical example of islet encapsulation was described in 2015 by Wertz and colleagues [30]. In this study, the ubiquitin-editing protein A20, encoded by TNFAIP3, is a negative regulator of immunostimulatory factors. Polyethylene glycol (PEG) hydrogel capsules loaded with A20-expressing islets are used as a drug release system to release immunosuppressants and growth factors to improve the state of transplanted islets. Once injected, the hydrogel can gel and provide support for the A20-expressing islets. The hydrogel shell of the capsules can promote the vascularization processes and prevents the immune system from attacking the pancreatic islets [31]. In order to protect the cells encapsulated in the hydrogel from being damaged by cytokines diffused into the capsules and to accelerate the vascularization processes, the researchers further added IL-1β, TNF-α, INF-γ, and other cytokines to modify the hydrogel. As a result, the cytokines can effectively protect the encapsulated cells against β cell-specific T lymphocytes and maintain glucose-stimulated insulin release from the islet cells [32].

Likewise, mesenchymal stem cells (MSCs) can release soluble cytokines and growth factors to neighboring cells to suppress the immune response, resulting in no local immunosuppression for MS [33]. In this context, MSCs and islet cells were simultaneously encapsulated into alginate hydrogel to improve the survival rate of the transplanted islet cells, promoting insulin secretion and new blood vessel formation [34,35].

Besides the pancreatic cells, the changes in the components of the encapsulation hydrogels can also change the destiny of the capsules. Studies have shown that the incorporation of tripeptide sequence Arg-Gly-Asp (RGD) in the islet-encapsulated PEG hydrogels could improve insulin response to glucose stimulation. Furthermore, the degradable hydrogel layer could enhance the vascular density at the graft site of the greater omentum, thereby improving the viability of encapsulated islets in a syngeneic diabetic rat model [36].

**Figure 3:**
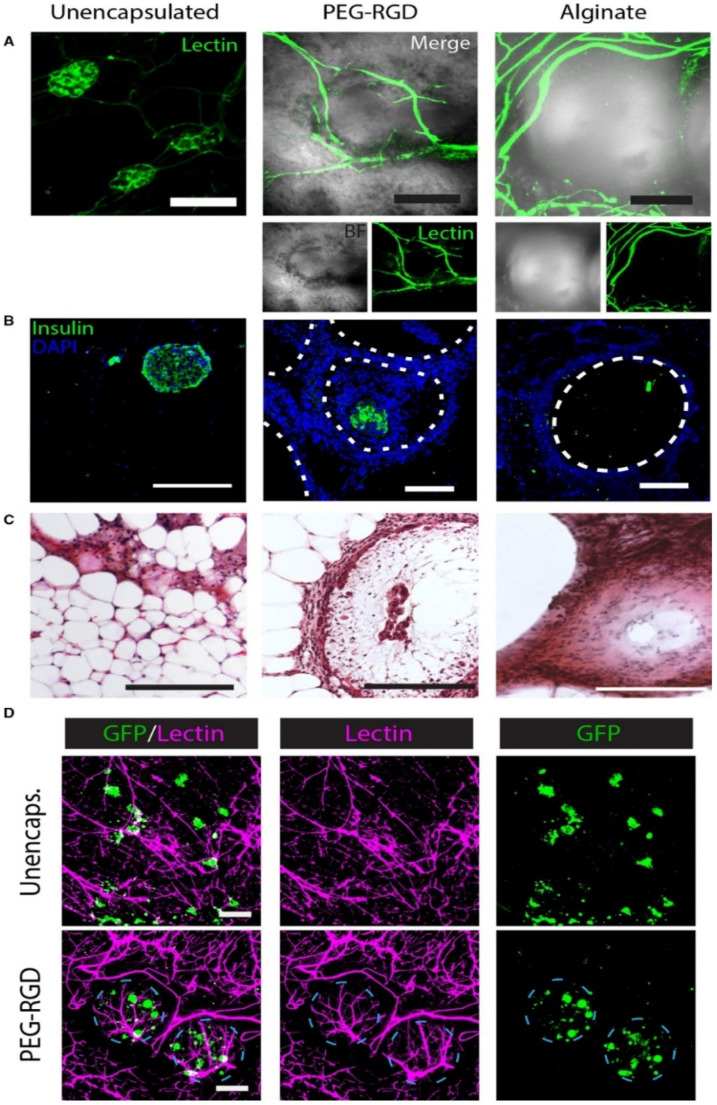
Histological evaluation of EFP-transplanted unencapsulated and encapsulated islets. (**A**) Lectin staining shows the vascular system of the graft; (**B**) IHC staining of the samples; (**C**) H & E staining of the samples; (**D**) Imaging of the PEG-RGD-encapsulated islets shows dense blood vessel formation on the surface of the microgels and living islets within the microgels. Reprinted with permission from Ref. [37] Copyright 2019, John Wiley and Sons.

To facilitate nutrient diffusion, researchers employed a microfluidic encapsulation system to enhance the insulin responsiveness of the encapsulated islets and allow the islets to engraft within the vascularized tissue space [37] (Figure 3). Pham et al. used surface modification technology, 3,4-dihydroxyphenethylamine (DOPA)-conjugated polylactide–polyethylene glycol nanoparticles carrying immune-suppressant FK506 (FK506/DOPA-NPs) (DOPA-NPs), and functionalized DOPA-NPs to form a multifunctional coating for antigen camouflage without interfering with islet viability and function. The coating effectively preserved the morphology and viability of the islets when co-cultured with xenogeneic lymphocytes for 7 days. The mean survival time of the islets coated with FK506/DOPA-NP was higher. This study suggests that the combination of surface camouflages and local low-dose immunosuppressive agents may prolong the survival time of the transplanted islets [38].

During the past few years, decellularized extracellular matrix (dECM), as a kind of natural polymer, contains various proteins, as well as growth factors, required for cell growth and differentiation, regulating biological balance with low toxicity and immunogenicity, has attracted much attention in biomedical fields [39,40]. Compared with polysaccharide- or protein/peptide-based materials, dECM-based materials can better mimic the ecological niche of natural tissues or organs [41]. In some cases, dECM plays a crucial role in particular tissue homeostasis, growth, and maturation, which makes it a special candidate for islet encapsulation with improved microenvironments [42].

Analogously, composite polymers for the formation of an interpenetrating network by complexing extracellular matrix (ECM) components of human-derived liposuction fluid with ionized gels of alginate matrices and heat-induced gels of pepsin-solubilized ECM pregels can achieve the in situ encapsulation of pancreatic islet cells (MIN6 β cells) [43,44]. Islets encapsulated in the microcapsules (≈640 µm), proliferated rapidly in vitro and displayed glucose-stimulated insulin responses due to the enhanced cell-matrix interactions [45]. When alginate was combined with the ECM-derived peptides, such as RGD, LRE, YIGSR, PDGEA, and PDSGR, islet dysfunctions due to the disruption of ECM interactions during the earlier islet isolation and side effects associated with immunosuppression can be overcome [46]. Porcine islets encapsulated in peptide-functionalized alginate microcapsules exhibit enhanced viability and glucose-stimulated insulin release. This study suggests that the ECM-derived peptides help to maintain the health of the encapsulated islets and may contribute to prolonging the lifespan of the encapsulated islet grafts [47].

In general, islet cell transplantation is one of the most promising treatments for type 1 diabetes, but the recipient’s immune response to the encapsulation polymers and cells is a major obstacle to the clinical application of islet cell transplantation. The control of the polymer component, thickness, and pore size around the islets is related to the level of mass exchange between the islets and the external small molecules and immunosuppression. Until the present, the traditional cell encapsulation techniques still have many limitations in clinical trials, with regard to islet cell protection effects, transplantation sites, and graft stabilities. The in-depth study of islet cell encapsulation materials, encapsulation strategies, and stem cell technologies is expected to improve the success rate of cell-based remedies.

## 4. Pancreas 3D Printing

3D bioprinting is a fully automatic layer-by-layer additive manufacturing process, which can deposit cells, growth factors, and other biomaterials through rapid prototyping (RP) technologies to fabricate bioartificial tissues and organs with multicellular components, hierarchical structures (especially branching vascular networks), and complex functions [48,49]. Currently, 3D bioprinting technologies have been successfully used to print many living tissues and organs [12], including blood vessels [50], skins [51], bones [52], cartilages [53], hearts [54], and livers [55]. Most of the 3D bioprinting technologies used for producing bioartificial pancreases belong to inkjet 3D printing, fused deposition modeling (FDM), extrusion-based 3D printing, and UV curing-based 3D printing [56,57,58,59,60,61]. The raw biomaterials for cell/growth factor-loading include natural polymeric solutions or hydrogels, synthetic polymeric solutions, and ECMs [62,63,64]. With these 3D bioprinting technologies and ‘bioinks’, all of the bottleneck problems which have perplexed tissue engineers, biomaterial researchers, pharmaceutists, and other scientists for several decades have been overcome sensibly [65]. These can be reflected in the following sections.

### 4.1. Natural Polymers for Pancreas 3D Printing

Natural polymers are macromolecular compounds that exist in nature, including proteins, polysaccharides, and their combinations, such as glycoproteins and proteoglycans [66,67]. Most of the natural polymers, such as gelatin, alginate, fibrinogen, and hyaluronic acid, are water-soluble, dissolving in inorganic solvents such as cell culture medium. The polymer solutions usually have good fluidities, excellent cytocompatibility, and can form water-rich hydrogels through the physical, chemical, and enzymatic cross-linking of the polymer molecules [68,69,70]. The water-rich hydrogels can not only embed living cells, growth factors, and other bioactive agents, transporting nutrients/oxygen to cells, but also discharge metabolic wastes produced by cells through the interpenetrating networks [71,72].

**Figure 4:**
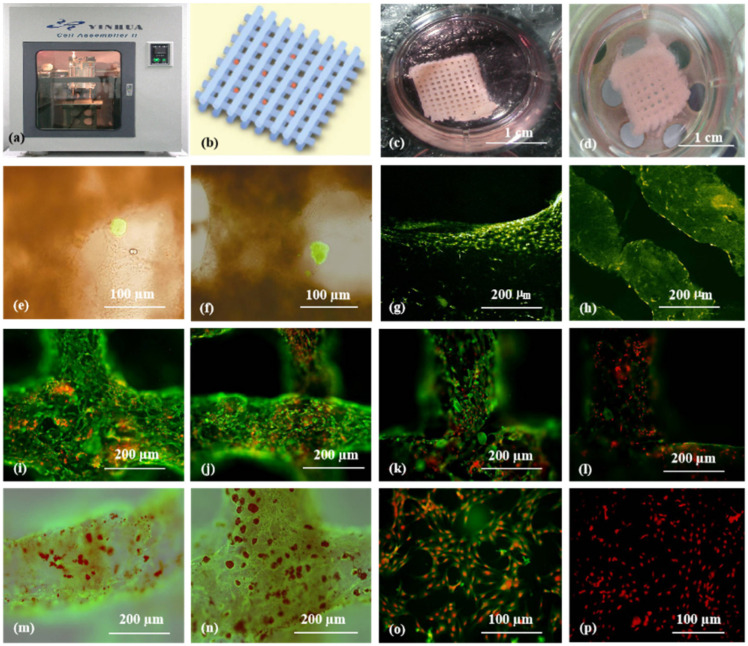
Cell-laden hydrogel constructs printed by Professor Wang: (**a**–**d**) grid gelatin/alginate/fibrin constructs containing ASCs and islets; (**e**,**f**) immunofluorescence staining of islet cells in the constructs; (**g**,**h**) immunofluorescence staining of ASCs differentiated into endothelial cells with EGF; (**i**–**l**) endothelial cell (green) immunostaining, nuclear (red) propidium iodide (PI) staining; (**m**,**n**) immunostained endothelial cells (green), and adipocytes (red) stained with oil red O; (**o**,**p**) immunostaining of two-dimensional (2D) cultured endothelial cells (green), differentiated from ASCs with pyridine iodide staining nucleus (red). Reprinted from Ref. [75].

As the main component of 3D printable ‘bioinks’, natural polymers have been widely used in pancreas 3D printing. The 3D printed islets embedded in natural hydrogels can maintain excellent biological activities with glucose regulation functions [73]. The first pancreas 3D printing technology was reported by Prof. Wang early in 2009 using gelatin/alginate/fibrin hydrogels [74] in which, adipose stem cells (ASCs), embedded in gelatin/alginate/fibrinogen solutions, and islets were printed into large-scale living organs with similar biological and physiological functions of their natural counterparts. When the pancreatic islets were deposited at designated locations with the ASC-laden gelatin/alginate/fibrin hydrogel, the ASCs can be induced to differentiate into vascular endothelial cells (ECs) and adipocytes dividually (or separately). The differentiation and self-organization of ASCs can be totally controlled by the growth factor combinations and the incorporated islets. This is a huge milestone in complex organ engineering/manufacturing areas, which has shown great potential in the establishment of physiological models of MS. When different drugs are applied to this model, the physiological responses are consistent with the in vivo experiments, suggesting that this model has strong advantages in high-throughput drug screening, pathological model establishment, as well as contributing to a better understanding of the multiple sclerosis pathogenesis of cells and drug development strategies [75] (Figure 4).

**Figure 5:**
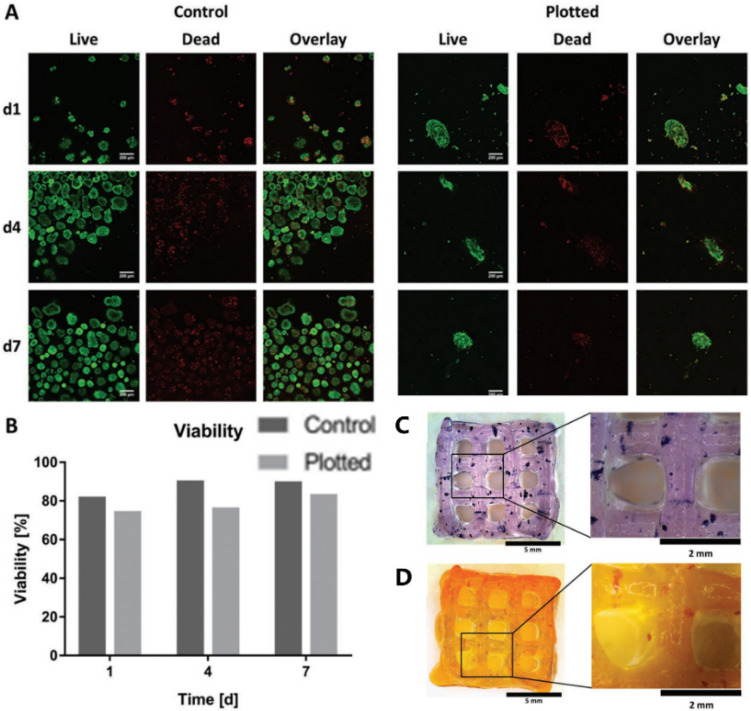
Islet viability assay and staining. (**A**) Live/dead staining of islets in Alg/MC gel (right) and free control islets (left). Live and dead cells are shown in green and red, respectively. (**B**) Semi-quantitative assessment of islet viability based on live/dead staining as shown in (**A**), *n* > 60 islets. (**C**) Islet-containing scaffolds were stained with thiazolyl blue tetrazolium bromide (MTT). (**D**) Islet-containing scaffolds were stained with dithizone (DTZ). Reprinted with permission from Ref. [76], Copyright 2019, John Wiley and Sons.

Later, in 2019, Duin et al. encapsulated islets into an alginate/methylcellulose hydrogel and constructed macroporous hydrogel structures via a simple 3D bioprinting technique. It was shown that the islets within the hydrogel had good viability and morphology and could continuously produce insulin and glucagon throughout the observation stage in responding to glucose stimulation [76] (Figure 5).

In 2021, Hu et al. developed a new ‘bioink’ based on natural alginate molecules. They added polymer Pluronic F127 to the alginate solution, which greatly improved the printability of the alginate-based hydrogel and the flexibility of the cross-linked structure. Meanwhile, hypomethylated pectin was added to reduce inflammation. The experimental results showed that the cellular constructs printed with pectin-alginate-pluronic ‘bioink’ could reduce tissue rejections by inhibiting TLR2/1 and ensure the survival of the insulin-producing β cells under inflammatory stress. It provides an improved strategy for the long-term survival of the transplanted islets in the treatment of type 1 diabetes [77].

To overcome the fundamental problems for islet or pancreatic cell transplantation, such as lacking adequate blood vessels in the constructs and allogeneic immune attack after implantation, the development of custom-designed bioartificial pancreases is urgently needed. This problem is expected to be solved using multi-nozzle 3D bioprinting technologies [78]. With the multiple nozzles, the distribution of many different cell types, including multicellular islets, can be controlled simultaneously to mimic the natural pancreas with the desired physiological functions.

### 4.2. Synthetic Polymers for Pancreas 3D Printing

**Figure 6:**
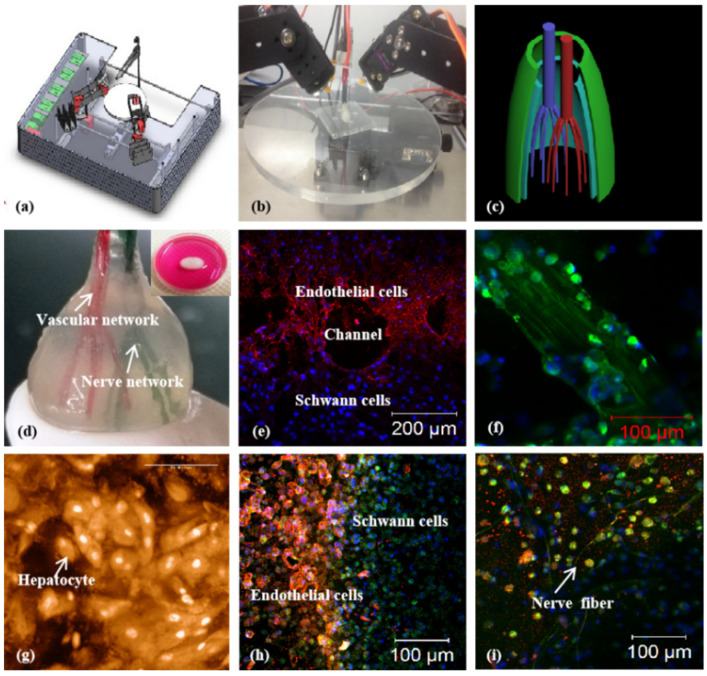
Vascularized and neuralized liver tissue models constructed by Professor Wang: (**a**,**b**) combined four nozzle printer; (**c**) a CAD model of the vascularized and neuralized liver tissue; (**d**) the 3D printed constructs containing vascularized and neuralized liver tissues; (**e**) immunofluorescence staining of endothelial cells and Schwann cells around the branching channels of the constructs; (**f**) nerve fibers formed in the 3D constructs; (**g**) hepatocytes in the 3D constructs, some of the cells in proliferation stage with two nucleus; (**h**) the interface between the endothelial cells and Schwann cells; (**i**) nerve fibers formed in the constructs. Reprinted from Ref. [75].

Synthetic polymers are artificially manufactured macromolecular compounds that cannot be obtained from nature. They are often obtained through a certain polymerization reaction, using small molecules called monomers, with known structures and relatively low molecular weights as raw materials [78]. Synthetic polymers are widely used in various fields such as electronics, automobiles, and transportation due to their excellent chemical and physical properties. Synthetic polymers, such as polylactic acid (PLA), polylactic-co-glycolic acid (PLGA), polyurethane (PU), and polycaprolactone (PCL) with good mechanical properties, in vivo histocompatibility, and structural stability, have been 3D printed widely as tissue engineering scaffolds for cell attachment and vascular/neural network building templates for organ implantation [75,79,80,81,82] (Figure 6).

Compared with natural polymers, most synthetic polymers have super mechanical properties, and 3D printed structures can be maintained in vivo for a long time [83]. For example, Song et al. printed a PLA structure by tuning the parameters of a low-cost 3D printer that could be accommodated by clusters of SC-β cells in a degradable fibrin gel. A finite element model of cellular oxygen diffusion consumption was used to determine the diameter of cell clusters to avoid severe hypoxia before vascularization. After the constructs were transplanted into mice, insulin was secreted in response to glucose injection, and the transplanted constructs maintained their structural integrity for 12 weeks. Unlike the pure cell encapsulation techniques, this approach could serve as a platform for advanced diabetes therapies using 3D printed cell replacements [84].

In another study, Farina presented a novel 3D printing and functionalized encapsulation system for the subcutaneous transplantation of pancreatic islets or islet-like cells. When the surface of the 3D printed PLA structure underwent some treatments, the hydrophilicity of the synthetic polymers was increased, which could facilitate cell attachment and proliferation. The implantation of a growth factor-rich platelet gel in a surface-treated encapsulation system could help to create a vascularized environment prior to loading human islets. Islets encased in this device could be protected from acute hypoxia and retain their function [85].

**Figure 7:**
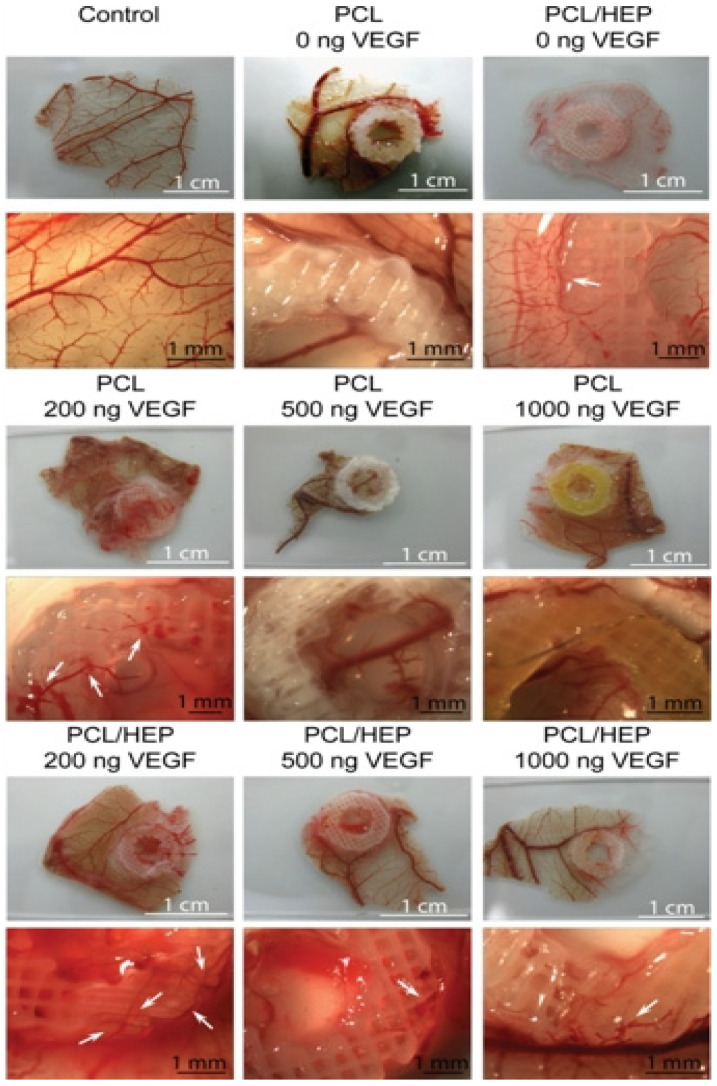
CAM assays were performed on PCL and heparin-coated 3D-printed PCL scaffolds with three different concentrations of VEGF. The 200 ng loading of VEGF induced stent formation with normal morphological vessels. Reprinted with permission from Ref. [86], Copyright 2016, Elsevier.

Similarly, Marchioli developed a PCL scaffold that could actively promote vascularization in extrahepatic islet transplantation. The PCL scaffold with a heparinized surface could electrostatically bind vascular endothelial growth factor (VEGF) to the alginate-encapsulated islets. Compared with the untreated PCL scaffold, heparin immobilization could increase the retention of the VEGF in the scaffold up to 3.6-fold. In a chicken chorioallantoic membrane model, the VEGF immobilized on the surface of the PCL scaffold could promote angiogenesis. After 7 days of implantation, the alginate-encapsulated islets exhibited functional responses to the glucose stimulation similar to the free-floating islets. The model has the potential to support rapid vascularization and islet endocrine function [86] (Figure 7).

### 4.3. ECM and dECM for Pancreas 3D Printing

As stated above, ECM is a macromolecular substance secreted by cells with a complex network, supporting, connecting, and regulating cell behaviors with the occurrence of tissue and organ formation [87,88]. An acellular matrix is a process of the decellularization of allogeneic tissue to remove antigenic components that can cause immune rejection, while completely retaining the 3D structure of the ECM with some growth factors, such as the fibroblast growth factor, VEGF, that play a significant role in stem cell differentiation [89,90]. Some of the ECMs have relatively good mechanical properties compared with the single natural polymeric hydrogels. Some of the ECMs demonstrate good histocompatibility and low immune rejection when they are implanted in the body [91,92].

**Figure 8:**
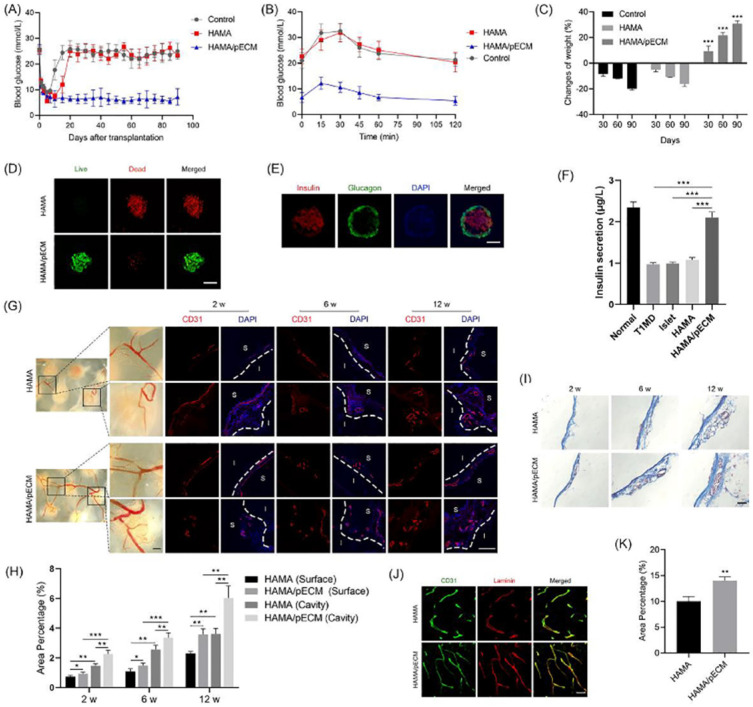
In vivo transplantation of the 3D printed pancreas-like organ. (**A**) Blood glucose level in the diabetic mice. (**B**) Intraperitoneal glucose tolerance test (IPGTT). (**C**) Weight changes of mice after the 3D printed pancreas-like organ transplantation. (**D**) Living and dead cell staining. (**E**) Insulin/glucagon/DAPI immunofluorescence images. (**F**) Comparison of serum insulin levels in different groups of mice. (**G**,**H**) CD31 immunostaining image and intensity comparison. (**I**) Masson trichrome staining images of the 3D printed pancreas-like organ. (**J**) Typical CD31 and layer adhesion immunostaining images of the 3D printed pancreas-like organ. (**K**) Percentage of angiogenesis. * *p* ≤ 0.05, ** *p* ≤ 0.01, *** *p* ≤ 0.001. Reprinted with permission from Ref. [95], Copyright 2022, Elsevier.

Similarly, an acellular extracellular matrix or dECM is a biological material derived from living organisms, and its 3D printed pancreas model is closer to the living environment of real islets, which is more conducive to the maintenance of islet function and the release of insulin [93]. In 2019, Kim et al. printed pancreatic-derived ECM (pdECM) for the creation of a native microenvironment for transplantable 3D pancreatic tissues. The results showed that the insulin secretion of human pluripotent stem cells and the maturity of insulin-producing cells were highly enhanced when they were cultured in the pdECM ‘bioinks’ and that the co-culture with human umbilical vein-derived endothelial cells could reduce the central islet necrosis under 3D culture conditions. The possibility of fabricating 3D islet structures with therapeutic graft dimensions was validated by the fusion with 3D bioprinting technology [93]. Hwang et al. developed a hybrid packaging system using 3D bioprinting technology, which consists of macroporous polymer capsules and nanoporous dECM hydrogels with islet-like aggregates. The exterior of the construct is designed as a go-through porous structure, β-cells can be encapsulated inside, and can maintain their activities with insulin secretion functions. The islet-like aggregates are formed through 3D bioprinting technology to improve cell vitalities and functions. The experimental results show that the hybrid packaging system has good biocompatibility, and the cells in the construct can connect through the go-through pores. These approaches are expected to solve the donor shortage problems to some degree and realize the clinical application of 3D printed pancreatic organs [94]. Wang et al. fabricated a novel ’bioink’ by combining pancreatic extracellular matrix (pECM) and hyaluronic acid methacrylate (HAMA) and used 3D printing technology to construct islet organoids. The islet cells maintained the biological functions in the structure through the Rac1/ROCK/MLCK signal pathway with improved bioactivities. When the pancreas structure was implanted into the diabetic model mice, the insulin level in the mice was significantly increased, and the blood glucose level in the mice remained at the normal level for 90 days. Compared with HAMA hydrogel, the HAMA/pECM hydrogel is more conducive to angiogenesis, and the blood vessel density is significantly increased, which brings hope for the construction of vascularized 3D pancreatic organs [95] (Figure 8).

Concisely, the most outstanding 3D bioprinting technologies and ‘bioinks’ for bioartificial ogan engineering/manufacturing are summarized in Table 1

## 5. Discussion

At present, great progress has been achieved in the field of cell encapsulation and 3D bioprinting. It has been proven that islet cells can maintain high activity and secrete insulin in most of the constructed models. Like other bioartificial organ engineering/manufacturing, there are still some unsolved issues to be explored in order to obtain an implantable bioartificial pancreatic organ [110,111].

Firstly, the bioartificial pancreases constructed from pure natural polymers and ECMs can hardly maintain their original shapes before the cells grow into mature pancreatic tissues [112], while the bioartificial pancreases constructed from pure synthetic polymers are difficult to load cells and bioactive agents. Therefore, material scientists need to develop new biomaterials or use a variety of composite materials to build a functional bioartificial pancreas with proper mechanical strengths and biological activities [113,114].

Secondly, it is better to use the patients’ own pancreatic cells or stem cell-derived pancreatic cells to build the implantable bioartificial pancreases for custom or personalized pancreas engineering/manufacturing and restoration. With the patients’ own pancreatic cells, most of the immunological rejection of the implantable bioartificial pancreases can be surmounted. A large amount of living cells with no immunogenicity is the guarantee for the organ-level replacement and respondence.

Thirdly, it is hard to construct precise structures that fully conform to the distribution of different cells in a natural pancreas. The pancreas contains a variety of adult cells, so mechanical engineers are required to design new 3D printers with multiple nozzles and high precision to deposit different cells in a predefined construct, mimicking their respective locations in natural pancreatic organs [115,116,117].

Fourthly, there is no such powerful equipment at present that can build a complex hierarchical vascular network, containing large arteries, branched arterioles, elaborate capillaries, and venous vessels (veins) integrally in a construct to maintain the nutrient transport and waste metabolism required inside the bioartificial pancreas [104,105]. Consequently, scientists need to design an induction strategy that can generate complex vascular networks in bioartificial pancreatic organs [118,119].

It is expected the 3D bioprinting pancreas will become the mainstream for future diabetes treatment. Problems such as the transportation of nutrients in the complex bioartificial organs, the formation of the branched hierarchical vascular networks, the biocompatibility of the 3D printed ‘bioinks’ in hosts, and the anti-suture/stress capabilities of the implanted bioartificial organs can be solved by more powerful updated 3D printers [120,121,122,123,124]. 3D bioprinting, as the most effective technology in the field of complex organ engineering/manufacturing, can fundamentally solve all of the problems faced by donor organ shortage and various MSs.

## 6. Conclusions

The construction of a clinically implantable bioartificial pancreatic organ requires the joint efforts of researchers in different fields, such as medicine, biology, materials, computers, engineering, chemistry, etc. Currently, 3D bioprinting, along with cell encapsulation technologies, has solved nearly all the bottleneck problems for bioartificial pancreas engineering/manufacturing. There are still some particular features, including an anti-sutural hierarchical vascular network with a full spectrum of blood vessels, that should be incorporated. With the rapid development of stem cells, biomaterials, and 3D printers, we can foresee that the 3D printing of bioartificial pancreases will save numerous patients in the future.

## Figures and Tables

**Table 1:** Outstanding 3D bioprinting technologies and polymers for bioartificial organ engineering/manufacturing.

Author (Year)	3D Bioprinting Technique	Polymer	Polymer Crosslinking Method	Result	Ref.
Yan et al. (2005, 2006)	Extrusion-based 3D bioprinting system with a single nozzle (Tsinghua University, China)	Gelatin, gelatin/alginate, or gelatin/chitosan	2.5% glutaraldehyde solution for gelatin, 10% calcium chloride (CaCl_2_) solution for alginate, 3% Na_5_P_3_O_10_ for chitosan	Living cells loaded in gelatin and/or gelatin/alginate hydrogels are first printed into large scale-up grid tissues with very high cell survivabilities (≥ 98%)	[55,96,97]
Xu et al. (2007)	Extrusion-based 3D bioprinting system with a single nozzle (Tsinghua University, China)	Gelatin/hyaluronate or gelatin/fibrinogen	Glutaraldehyde solution for gelatin, 3% Na_5_P_3_O_10_ for chitosan	Large scale-up grid tissues are with very high cell survivabilities (100%)	[98,99]
Xu et al. (2007, 2008)	Low-temperature extrusion-based 3D bioprinting with one or two nozzles (Tsinghua University)	Gelatin, gelatin/alginate, gelatin/fibrinogen, PLA, PLGA, or PU	Glutaraldehyde solution for gelatin, CaCl_2_ solution for alginate, Na_5_P_3_O_10_ for chitosan	Anti-sutural vascular network is formed using synthetic PU, cell cryopreservation agents are used for cell-laden hydrogel 3D printing	[79,100]
Li et al. (2009)	Extrusion-based 3D bioprinting system with two nozzles (Tsinghua University, China)	20% gelatin, 30% gelatin/5% alginate/10% fibrinogen (2:1:1), and/or gelatin/alginate/chitosan	Double crosslinking alginate/fibrinogen with CaCl_2_ and thrombin solutions; triple crosslinking alginate/fibrinogen/chitosan with CaCl_2_/thrombin/Na_5_P_3_O_10_ solutions	Two types of living cells loaded in gelatin/alginate and/or gelatin/alginate/fibrinogen hydrogels are first printed into large scale-up organs with a predesigned hierarchical vascular network	[66,74,101,102]
Sui et al. (2009, 2010)	Extrusion-based 3D bioprinting and cell cryopreservation techniques are combined comprehensively (Tsinghua University, China)	Gelatin/alginate hydrogels with 5% dextrain-40	CaCl_2_ solution for alginate	The physical and chemical properties of the hydrogels are totally changed which is beneficial for long-stem storage of the bioartificial tissues/organs	[103,104]
Cui et al. (2009–2013)	Low-temperature extrusion-based 3D bioprinting with one or two nozzles (Tsinghua University)	PU, PLGA, gelatin/alginate/fibrinogen, and/or gelatin/alginate/chitosan	CaCl_2_ solution for alginate, thrombin solution for fibrinogen, Na_5_P_3_O_10_ for chitosan	Natural and synthetic polymer systems are printed together with anti-sutural PU (or PLGA) and long-term storable cell/hydrogels	[105,106,107]
Zhao et al. (2015–2016)	3D bioprinting with multiple nozzles (Tsinghua University)	PU, PLGA, gelatin/alginate/fibrinogen, and/or gelatin/alginate/chitosan	CaCl_2_ solution for alginate, thrombin solution for fibrinogen, Na_5_P_3_O_10_ for chitosan	Natural and synthetic polymer systems are printed together with anti-sutural PU (or PLGA) and long-term storable cell/hydrogels	[108,109]
Duin et al. (2019)	GeSiM mbH (Radeberg, Germany)	Alginate and methylcellulose	CaCl_2_ solution for alginate	The islets can continuously produce insulin and glucagon in the structure	[76]
Hu et al. (2021)	Biobots 1 desktop 3D bioprinter (Philadelphia, PA, USA)	Pectin, alginate, and Pluronic	CaCl_2_ solution for alginate	The structure supports the survival and function of islet β cells and has the capability of immune regulation	[77]
Song et al. (2016)	The single-extruder 3D printer Makergear M2 (Makergear; M2 3D Printer—Assembled)	PLA and fibrinogen	Thrombin solution for fibrinogen	Cells grow well in the 3D printed constructs	[84]
Farina et al. (2017)	A fused deposition method (FDM) based 3D printer Replicator™ 2× (MakerBot Industries, Houston, TX, USA.)	PLA	No applied	The encapsulation system can produce sufficient and rapid graft vascularization and can increase the vitality and function of the pancreatic islets	[85]
Marchioli et al. (2016)	An extrusion-based additive manufacturing machine (sysENG, Salzgitter Flachstöckheim, Germany)	PCL and alginate	CaCl_2_ solution for alginate	The developed platform has the potential to support rapid vascularization and islet endocrine function	[86]
Wang et al. (2022)	UV curable printing (Affiliated Hospital of Nantong University, Nantong, China)	Hyaluronic acid methacrylate and pancreatic extracellular matrix	UV for hyaluronic acid methacrylate	The 3D structure can promote the generation of the vascular network, and the islet cells in the construct can maintain the blood glucose level in mice at a normal level	[95]

## Data Availability

Not applicable.

## Abbreviations

3D	three-dimensional
CAD	computer-aided design
PEGDA	PEG-diacrylate
PEG	polyethylene glycol
MSCs	mesenchymal stem cells
PLGA	polylactic-co-glycolic acid
dECM	decellularized extracellular matrix
ASCs	adipose stem cells
MS	metabolic syndrome
ALG	alginate
Gel	gelatin
PLA	polylactic acid
PU	polyurethane
ECs	endothelial cells
EGF	epidermal growth factor
PI	propidium iodide
IBMX	isobutylmethylxanthine
2D	two-dimensional
DTZ	DTZ
PCL	polycaprolactone
VEGF	vascular endothelial growth factor
ECM	extracellular matrix
HAMA	hyaluronic acid methacrylate
pECM	pancreatic extracellular matrix
IPGTT	intraperitoneal glucose tolerance test
GelMA	gelatin-methacryloyl
